# Influence of La^3+^ Substitution on Structure, Morphology and Magnetic Properties of Nanocrystalline Ni-Zn Ferrite

**DOI:** 10.1371/journal.pone.0170075

**Published:** 2017-01-12

**Authors:** Y. K. Dasan, B. H. Guan, M. H. Zahari, L. K. Chuan

**Affiliations:** Department of Fundamental and Applied Sciences, Universiti Teknologi PETRONAS, Bandar Seri Iskandar, Tronoh, Perak Darul Ridzuan, Malaysia; Universiti Malaysia Perlis, MALAYSIA

## Abstract

Lanthanum substituted Ni-Zn ferrite nanoparticles (Ni_0.5_Zn_0.5_La_x_Fe_1-x_O_4_; 0.00 ≤x≤ 1.00) synthesized by sol-gel method were presented. X-ray diffraction patterns reveal the typical single phase spinel cubic ferrite structure, with the traces of secondary phase for lanthanum substituted nanocrystals. In addition, the structural analysis also demonstrates that the average crystallite size varied in the range of 21–25 nm. FTIR spectra present the two prominent absorption bands in the range of 400 to 600 cm^-1^ which are the fingerprint region of all ferrites. Surface morphology of both substituted and unsubstituted Ni-Zn ferrite nanoparticle samples was studied using FESEM technique and it indicates a significant increase in the size of spherical shaped particles with La^3+^ substitution. Magnetic properties of all samples were analyzed using vibrating sample magnetometer (VSM). The results revealed that saturation magnetization (M_s_) and coercivity (H_c_) of La^3+^ substituted samples has decreased as compared to the Ni-Zn ferrite samples. Hence, the observed results affirm that the lanthanum ion substitution has greatly influenced the structural, morphology and magnetic properties of Ni-Zn ferrite nanoparticles.

## Introduction

Ferrite materials of spinel structural formula AB_2_O_4_ are mainly composed of about 70% iron oxide (Fe_2_O_3_) and about 30% of other metal oxides including MgO, MnO, NiO, CuO, and or FeO [1, 2]. Metal oxide nanoparticles have been intensively studied both theoretically and practically due to the electric, dielectric, optical and magnetic properties that are significantly different as compared to the bulk materials [3, 4]. Generally, the physical properties of ferrite materials are governed by processing techniques, stoichiometry, and also the dispersion of cations among tetrahedral-A sites and octahedral-B sites. The preference of cation to occupy either A, or B sites are dependent on their ionic radius, crystal field, electronic configuration and also the ionic polarization [5–9].

Ni-Zn spinel ferrites nanoparticles found to be one of the versatile ferrites from the view of technological applications with high resistivity and low eddy current loss [10]. Zinc ferrite bulk material possesses normal spinel structure with their divalent and trivalent cations are located on the tetrahedral and octahedral sites respectively. On the other hand, Nickel ferrite bulk materials have a spinel structure, where the trivalent cations occupy the A and B sites equally while all the divalent cations move to the octahedral site. Generally, this type of ferrites is called as a hopping semiconductor, where their conducting properties are the thermally activated hopping of electrons from one to another cation [10]. There is numerous attempt have been made in order to enhance the qualities of ferrites by incorporating the same suitable nonmagnetic/diamagnetic impurities with different valence state at the A and B sites includes Copper [11, 12], Manganese [13], Praseodymium [14], Lanthanum [15], Neodimium [16] ion and etc [17]. Lanthanum is known as the second most abundant and lightest rare earth element (REE) in the lanthanide series. This silvery white mineral found in monazite and bastnasite ores. Lanthanum possesses distinct quality as compared to other REE such as simple electronic spectra which is helpful for experimental analysis; it has the highest boiling point and lowest vapour pressure at its melting point; and at atmospheric pressure lanthanum is the only superconducting REE [18]. Therefore, lanthanum is demand for some important application includes a) used in the manufacture of expensive glasses as lanthanum imparts a high refractive index to the glass [19]; b) used in NiMH batteries that are currently used in almost all hybrid-electric vehicles [20, 21]; c) Lanthanum rich compounds are used in alloy and fluid cracking catalyst for petroleum refinery industry [22]

Rare earth materials are known to possess good electrical insulation properties with high electrical resistivity. Therefore, the substitution of these rare earth ions into spinel ferrites could alter the electrical and magnetic properties. Moreover, these rare earth ions have a huge influence on the magnetic anisotropy of the system making the spinel ferrite as promising materials replacing the hexaferrite or garnets [16, 23]. The partial substitution of Fe^3+^ ion by rare earth materials ion into the spinel ferrites structure leads to a structural distortion and induce strain, hence modifies the magnetic and electrical properties remarkably [24–26]. Generally, the rare earth ions occupy the octahedral B-sites with limited solubility in the spinel lattice structure due to their large ionic radius. However, based on the previously reported work the behavior of different rare earth ions are varied in spinel different ferrites and yield different structural properties [27].

Various methods have been employed in order to synthesize the soft spinel ferrite materials Including chemical co-precipitation [28], hydrothermal method [29], mechano-chemical method [30], microemulsion method [28], rheological phase reaction method [31], and also sol-gel method [7]. Wu et al. [32] synthesized Ni _0.5_Zn _0.5_La_x_Fe_2–x_O_4_ using solid-state reaction at low temperatures for the first time. The results showed that the calcination temperature affects the magnetic properties of lanthanum substituted Ni-Zn ferrite, while the highest coercivity value of 120.09 Oe obtained at 800°C of calcination temperature. Ahmed et al. [10] have examined the structural and electrical properties of La^3+^ substituted Ni-Zn ferrite prepared by standard ceramic method. The obtained results showed an appearance of small peaks which represents the secondary phase due to the addition of lanthanum ion. Wang et al. [15] prepared Ni_0.8_Zn_0.2_La_x_Fe_2−x_O_4_ nanomaterial by sol gel method. The structural and morphological nalaysis of obtained samples shows that the addition of lanthanum ion has reduced the grain size. Further, lanthanum ion substitution has decreased the saturation magnetization (M_s_) from 69.81 to 46.75 emu/g. Among these techniques, sol-gel synthesis has been receiving much attention as they can be applied to an extremely wide variety of materials and also they offer the possibility of controlling the size, shape, and distribution of particles [33].

To the best of our knowledge, there aren't many research works focused on the detailed structural and morphological analysis of the lanthanum ion substitution into the spinel ferrite materials. Therefore, the current work is intended on investigating the effect of rare earth lanthanum ion substitution and distribution within the A and B sublattices on the structural, magnetic (super exchange interactions and spin alignment) and morphological properties of Ni-Zn ferrite nanoparticles. The structural, magnetic and morphological properties of synthesized nanoparticles were examined using XRD, FTIR, FESEM and VSM analysis.

## Experimental

All chemicals used were of analytical grade with purity ≥ 99% and all the stock solution was prepared with distilled water. Ferric nitrate Fe(NO_3_)_3_.9H_2_O, Citric acid C_6_H_8_O_7_, Nickel nitrate Ni(NO_3_)_3_.6H_2_O, Zinc nitrate Zn(NO_3_)_2_.6H_2_O, Lanthanum nitrate La(NO_3_)_3_.6H_2_O were used as the starting materials. La^3+^ substituted Ni-Zn ferrite having the general chemical formula of Ni_0.5_Zn_0.5_La_x_Fe_1-x_O_4_, where x = 0.0, 0.1, 0.2, 0.4, 0.6, was prepared through a typical sol-gel method as previously reported [34–36]. The nitrate salts of all the necessary elements for the final sample was used in stoichiometric amounts and were dissolved in 1M citric acid (C_6_H_8_O_7_) solution. The mixture was stirred with heating at 80°C for 24 hours in order to facilitate the gelation process. The resulting thick gel was then further dried in an oven at 110°C for another 24 hours. The resulting dried gel was then crushed into fine powders before being subjected to calcination at 900°C for 3 hours to complete the ferrite synthesis process. Citric acid acts as an organic fuel during the combustion by providing a platform for initiating the redox reactions between the reactants owed to its high heat of combustion [37].

The crystal structure of Ni_0.5_Zn_0.5_La_x_Fe_1-x_O_4_ ferrite powder was characterized by x-ray diffraction (XRD) analysis by using a Bruker D8 powder x-ray diffraction system with Cu-Kα radiation (λ = 1.5418 Å) at scanning angles 10°-90°. Structural information such as the present phases, lattice parameter, and crystallite size were able to be extracted from the samples’ diffraction patterns. The microstructure and morphology of substituted ferrite nanoparticles was examined using field emission scanning electron microscopy (FESEM model Zeiss Supra VP55) with an acceleration voltage of 20 kV. Further, fourier transform infrared spectroscopy (FTIR) was taken on Perkin-Elmer infrared spectrometer within the wave number range of 400–4000 cm^-1^. Magnetic measurements were performed by employing the vibrating sample magnetometer (VSM) with an applied field of 20 kOeto reach saturation and the magnetic hysteresis loops were measured at room temperature.

## Results and Discussion

A Ni_0.5_Zn_0.5_La_x_Fe_1-x_O_4_, (x = 0.0, 0.1, 0.2, 0.4, 0.6) nanocrystals was synthesized through a simple sol-gel method. The structural, morphological, and magnetic properties of Ni-Zn-La ferrite nanocrystals were examined for different composition of lanthanum in Ni-Zn ferrite nanocrystals. Further, the lanthanum ion substitution and distribution within the A and B sublattices of Ni-Zn ferrite nanocrystals was determined based on these observations.

### XRD analysis

**Fig 1:**
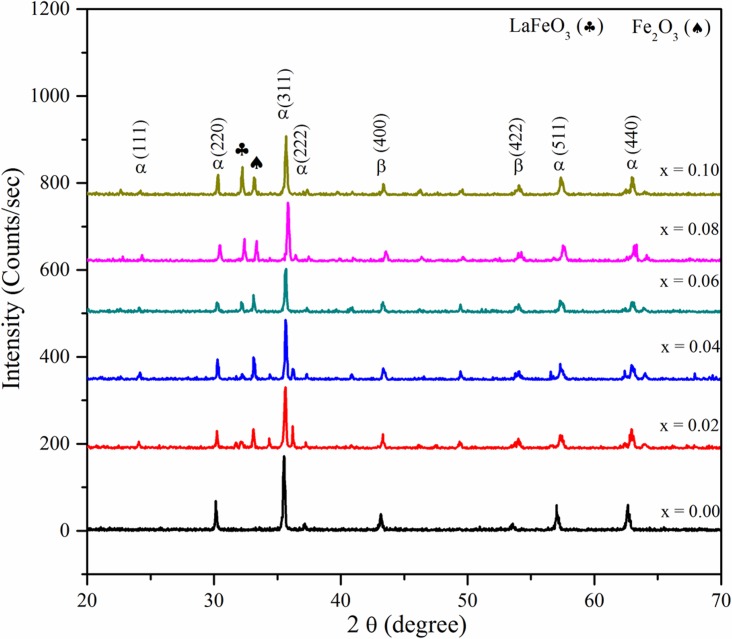
Structural and grain size analysis of La^3+^ substituted Ni-Zn ferrite nanoparticle. XRD diffraction patterns for the sample of Ni_0.5_Zn_0.5_La_x_Fe_1-x_O_4_ nanocrystalline (α: Ni-Zn ferrite; β: iron oxide) at various La+3 substituted concentration.

XRD patterns of Ni_0.5_Zn_0.5_La_x_Fe_1-x_O_4_ nanocrystals, for all the samples with x = 0.00, 0.02, 0.04, 0.06, 0.08, and 0.1 are shown in Fig 1. The X-ray diffraction patterns reveal a single phase cubic spinel structure with few traces of secondary phase. Furthermore, the observed diffraction peaks could be assigned to the reflection plane of (111), (220), (311), (400), (422), (511) and (440) which could be indexed to a single-phase Ni-Zn ferrite nanocrystal (α-phase). Besides that, the iron oxide (β-phase) was also detected in XRD pattern [28, 38, 39]. Meanwhile, the peak corresponding to 2θ = 32.21° (200) is attributed to secondary phase at the grain boundaries for LaFeO_3_ (♣) (ICDD PDF #74–2203) except for the cubic spinel phase. The intensity of LaFeO_3_ peak has increased with the increase in La^3+^ ion concentration. Furthermore, another secondary phase of Fe_2_O_3_ (♠) 2θ = 33.22° (ICDD PDF #86–2368) was detected with an increase in lanthanum ion concentration [40–43]. The appearance of secondary phase of a) LaFeO_3_ (♣) in the Ni-Zn-La ferrite nanoparticles demonstrates that La^3+^ ion substitution has a solubility limit in the spinel lattice; b) Fe_2_O_3_ (♠) in the Ni-Zn-La ferrite nanoparticles shows that there was more than fifty mole percentage of the standard iron oxide content in the formula of spinel ferrite [38].

The lattice parameter of individual composition was investigated by using the following relation:

$$a=d_{hkl}\;\sqrt{h^{2}+k^{2}+l^{2}}$$ (1)

Where, ‘ɑ’ is the lattice constant, d is the inter-planar distance and (h, k, l) are the Miller indices [44].

Whereas the average crystallite diameter of the powder estimated from the intense (311) peak of XRD diffraction pattern using the Scherrer’s Eq (2):

$$D=\frac{0.9\lambda }{B\;Cos\;\theta }$$ (2)

Where D is the average crystalline dimension perpendicular to the reflecting phases, λ is the wavelength of the X-ray source which is taken as 1.5406 Å, and B is the full width at half maximum (FWHM) and θ is the Bragg’s angle [45, 46]. The calculated values for lattice constant, planner distance and crystallite size at (311) peaks are tabulated in Table 1.

**Table 1:** Lattice parameters, interplane distance and crystallite size of Ni_0.5_Zn_0.5_La_x_Fe_1-x_O_4_ nanocrystalline.

Samples	Lattice constants	Interplane Distance	Crystallite Size (nm)
	a (Å)	(d Å)	
X = 0.00	8.557	2.528	22.42
X = 0.02	8.357	2.520	23.30
X = 0.04	8.347	2.517	24.22
X = 0.06	8.351	2.518	24.41
X = 0.08	8.308	2.505	22.76
X = 0.10	8.345	2.516	21.05

The calculated crystallite size of Ni_0.5_Zn_0.5_La_x_Fe_1-x_O_4_ nanocrystals for this work is found to be smaller than the previously reported work where the crystallite size for the lanthanum substituted nanocrystals ranged from 22 nm to 24 nm. The calculated values in Table 1, show that the crystallite size of prepared ferrite samples increases with an increase in the amount of La^3+^ ions concentration up to X = 0.06, due to the larger ionic radii that that of Fe^3+^ ions which induces the grain growth [47, 48].

However, Table 1 shows the gradual decrease in the crystallite size value with an increase in La^3+^ substitution at above X ≥ 0.06 which is in agreement with the results observed in the literature [49]. There are two possible explanations to account for this decrease in crystallite size with an increase in La^3+^ substitution concentration, a) Firstly, the large size mismatch between La^3+^ and Fe^3+^ induces the crystalline anisotropy during the substitution of ions which creates the strain inside the volume of the crystals with an increase in La^3+^ substitution. Furthermore, by balancing the crystal anisotropy and volume strain to each other the present system could remain in stable equilibrium. Therefore, the crystallite size decreases with an increase in La^3+^ substitution concentration in order to reduce the volume strain; b) Secondly, in the crystal structure of La^3+^ substituted Ni-Zn ferrite, Zn ^2+,^ and Ni prefers tetrahedral (A-site) and octahedral (B-site) sites respectively. On the other hand, Fe^3+^ occupies either tetrahedral or octahedral site while La^3+^ ions have a tendency to enter into the octahedral site. However, it is difficult for lanthanum ion to substitute Fe^3+^ as La^3+^ has much larger ionic radius than that of Fe^3+^ and requires more activation energy to enter octahedral sites as the bond energy of La-O is higher as compared to Fe-O. Therefore, instead of occupying the Fe^3+^ sites in the lattice the La^3+^ enter the lattice at interstitial sites due to extra stress coming from partial lanthanum ion which suppresses the crystallization of Ni-Zn ferrites [50, 51].

There is slight changes were observed for lattice constant ‘*a*’ which could be associated with the increasing La^3+^ content where the ionic radius of La^3+^ (1.6061 Å) is bigger than that of Fe^3+^ ion (0.645 Å) replacing iron ions on octahedral B-site which causes asymmetry in the structure. Hence, the lattice constant should be aggrandized with the increasing content of the La^3+^ during the substitution process [15, 52]. Besides that, the decrease in lattice constant with the increase of La^3+^ content could be attributed to the compression of spinel lattice induced by the secondary phases due to the difference in thermal expansion coefficients [42, 53]_._

### FTIR analysis

**Fig 2:**
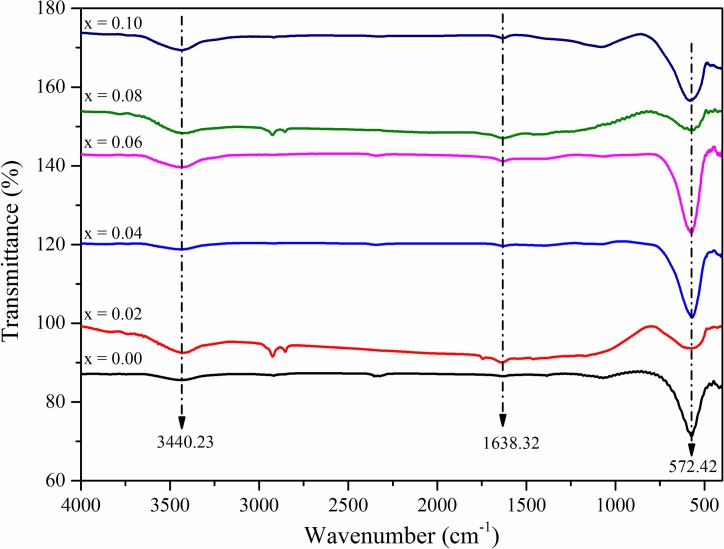
FTIR spectroscopic analysis to identify the types of chemical bonding in La^3+^ substituted Ni-Zn ferrite nanoparticle. FTIR spectra for the samples of Ni_0.5_Zn_0.5_La_x_Fe_1-x_O_4_ nanocrystalline at various La^3+^ substitution concentrations.

Fig 2. Show the FTIR spectra of the investigated Ni_0.5_Zn_0.5_La_x_Fe_1-x_O_4_ nanocrystalline samples at increasing La^3+^ concentration. FTIR spectra demonstrate the presence of two fundamental absorption bands which are attributed to the normal and inverse cubic spinel ferrites. Generally the higher frequency absorption band ‘ν_1_’ appears in the range of 500 to 600 cm^-1^ represents the intrinsic vibration of tetrahedral group, while the octahedral groups are represented by the lower frequency absorption band ‘ν_2_’ in the range of 350–490 cm^-1^ [54, 55]. Since the tetrahedral site dimensions are less as compared to the octahedral, the band frequency of ‘ν_1_’ is higher than the ‘ν_2_’ absorption band [56]. The characteristic bands that appear at wavenumber 572.42 cm^-1^ attributed to the metal-oxygen stretching vibration of Fe^3+^-O^2^ [57]. Further, the spectrum shows absorption bands at 1638.32 cm^-1^ corresponding to NO_3_^-1^ ions, and also due to the presence of carboxyl group (COO-) [46]. The broadband that appears at 3440.23 cm^-1^ affiliated to hydrogen bonded O-H stretching vibration [58]. The difference in intensity of ‘ν_1_’ and ‘ν_2_’ absorption bands is due to the changes in the bond length of Fe^3+^-O^2^ at tetrahedral A-sites and octahedral B-sites [2, 4].

It can be observed from the IR spectrum that the absorption band ‘ν_1_’ intensity increases with an increase in La^3+^ ion substitution concentration, while band ‘ν_2_’ does not show any changes. The variation in the intensity of ‘ν_1_’ is caused by the nature of lanthanum ion which prefers to occupy the octahedral B-site and forces the iron ion into A-site with an increase in the La^3+^ concentration, which increases the radius of B-site. However, with an increase in lanthanum ion concentration above X≥ 0.06 leads to decrease in the ‘ν_1_’. Additionally, it is known that the variation in the position of IR spectra peaks is mainly dependent on the amount of substituent, method of preparation, grain size and also the density of particles. Moreover, the intensity is the ratio of change of dipole moment with the inter-nuclear distance (dμ/dr) [4, 59].

### Morphology analysis

**Fig 3:**
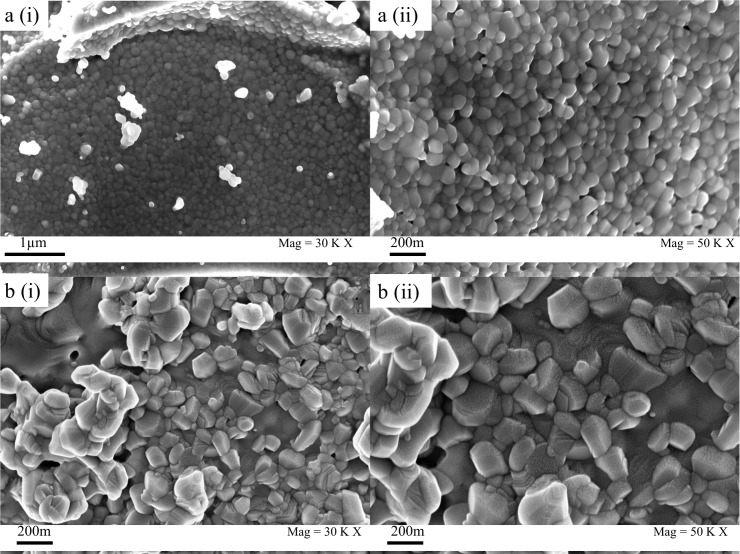
Morphological analysis of La^3+^ substituted Ni-Zn ferrite nanoparticle. FESEM micrographs of typical a) Ni-Zn ferrite (i) 30KX (ii) 50KX and b) Ni_0.5_Zn_0.5_La_x_Fe_1-x_O_4_ at (x = 0.02) (i) 30KX (ii) 50KX.

The FESEM images of Ni_0.5_Zn_0.5_La_x_Fe_1-x_O_4_ nanoparticles (0.0≤ X ≤0.10) are shown in Fig 3. Microstructure of Ni-Zn ferrite (X = 0.00) exhibits an angular morphology. Meanwhile, the microstructure of the particles became irregular in size and shape with La^3+^ substitution [60]. The particles tend to agglomerate as they encounter a permanently magnetized moment proportional to their volume. Moreover, the high calcination temperature can also attribute to the agglomeration of particles due to emerging forces including Van-der walls, capillary and electrostatic forces which create mutual interaction between particles [61, 62]. It can be observed from Fig 3 that the agglomeration is increasing with an increasing lanthanum ion concentration [48, 63]. Besides that, the larger ionic radius of lanthanum ion could attribute to the increase in grain size which occupy on the grain boundary on the outlay of cations vacancies in the lattice [64]. However, the average crystallite sizes of the substituted ferrite samples obtained from XRD analysis are significantly smaller than the values determined by FESEM analysis. The differences in average crystallite size of nanoparticles are associated with the fact that the value observed by FESEM analysis has the size of the secondary particles, where they are composed of several crystallites by the soft reunion. Moreover, the X-ray line broadening analysis reveals only the size of a single crystallite [32].

The composition of elements (%) and their atomic percentage of substituted ferrite nanoparticles were measured by elemental diffraction spectrum (EDS). Table 2 confirms the presence of Fe, Ni, Zn, La, and O in the sample which is used to further validate that lanthanum doped into Ni-Zn ferrite nanocrystals. Furthermore, the recorded values in Table 2 shows a gradual increase in Lanthanum composition which is in line with the synthesis condition. On the other hand, the detected traces of Carbon are mainly due to the coating applied to the sample prior to testing [65].

**Table 2:** The composition of elements of Ni_0.5_Zn_0.5_La_x_Fe_1-x_O_4_ nanocrystals at various La^3+^ substitution concentrations.

Elements (K)		X = 0.02	X = 0.04	X = 0.06	X = 0.08	X = 0.10
**C**	Weight (%)	5.90	-	-	-	-
	Atomic (%)	16.07	-	-	-	-
**O**	Weight (%)	20.99	20.70	21.65	26.75	21.85
	Atomic (%)	42.94	49.08	50.56	57.89	51.52
**Fe**	Weight (%)	49.05	51.01	51.73	42.90	44.20
	Atomic (%)	28.74	34.64	34.61	26.60	29.86
**Ni**	Weight (%)	9.93	11.11	13.55	14.82	13.53
	Atomic (%)	5.54	7.18	8.62	8.74	8.69
**Zn**	Weight (%)	12.77	14.36	8.89	10.34	14.34
	Atomic (%)	6.39	8.33	5.08	5.47	8.28
**La**	Weight (%)	1.35	2.82	4.18	5.20	6.08
	Atomic (%)	0.32	0.77	1.12	1.30	1.65

### Magnetic property

**Fig 4:**
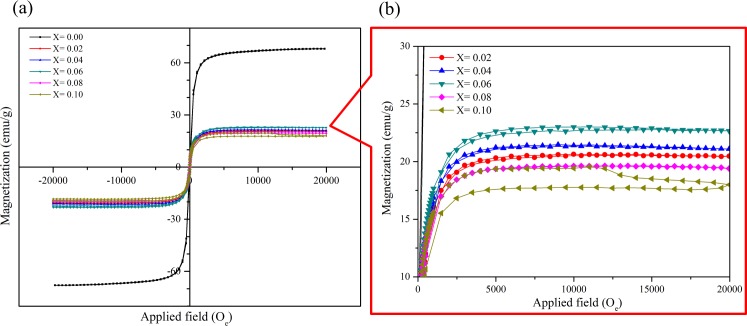
Magnetic properties of La^3+^ substituted Ni-Zn ferrite nanoparticle hysteresis loops. Hysteresis loops of (a) Ni_0.5_Zn_0.5_La_x_Fe_1-x_O_4_ nanocrystals at various La^3+^ substitution concentrations (b) Enlarged view of hysteresis loop.

**Fig 5:**
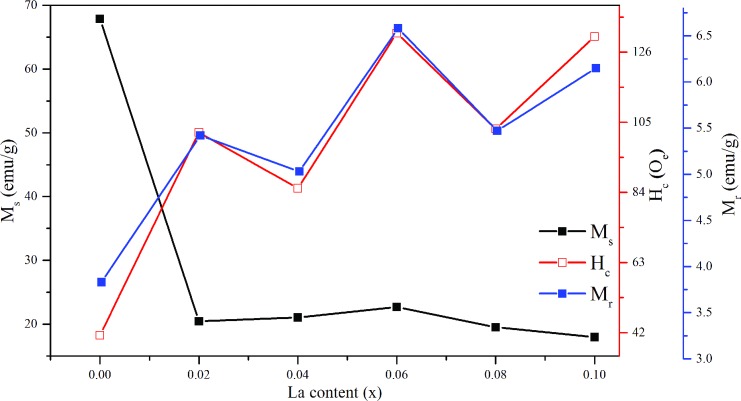
Magnetic properties of La^3+^ substituted Ni-Zn ferrite nanoparticle p. Variation of saturation magnetization (M_s_) coercivity (H_c_) and remanence (M_r_).

The magnetic hysteretic loop of Ni_0.5_Zn_0.5_La_x_Fe_1-x_O_4_ nanocrystals (0.0≤ X ≤0.10) is given in Fig 4. The measurements for magnetic parameters such as magnetization saturation (Ms), coercivity (Hc) and remanence (Mr) were analyzed based on this hysteresis loop and presented in Fig 5. It can be observed from the Fig 4. that the value of saturation magnetization (M_s_) for Ni-Zn ferrite sample is very high as compared to the La^3+^ ion substituted samples due to high crystallinity and uniform morphologies which can be observed from Fig 3. This is also due to the substitution of nonmagnetic lanthanum ion which leads to the formation of the nonmagnetic spinel structure. In addition to this, the substitution of La^3+^ ion causes the transformation of the collinear ferromagnetic order into non-collinear arrangement and disintegration of ferromagnetic order that reduces the M_s_ [50].

As the lanthanum ion concentration increases (X = 0.02–0.06) in Ni_0.5_Zn_0.5_La_x_Fe_1-x_O_4_ nanocrystals, the saturation magnetization shows a gradual increase due to the increase in the crystallite size and minimization of the surface effect [66]. However, the saturation magnetization decreases with further increase in lanthanum concentration at above X ≥ 0.06. The decrease in saturation magnetization can be explained based on two factors: (a) crystallite size and (b) site occupancy of the cations plus the modification in the super exchange effects caused by substituting La^3+^ [66]. As the crystallite size decreases (X ≥ 0.06), the ratio of surface to volume atom increases which results in the prominent surface effect. Surface atoms are under the effect of the strain due to the distorted surface structure thus creates vacancies, a variety of the interatomic spacing and low coordination numbers. As a consequence, these factors could induce broken exchange bonds for the surface atoms which results in spin disorder. The disordered spins at the surface demonstrate low magnetization. Furthermore, the disordered spins increase with the decrease in crystallite size, hence reduces the saturation magnetization [66, 67].

In addition, the observed reduction of the saturation magnetization can be described based on the cation dispersion and exchange interaction between iron and between lanthanum ions at tetrahedral A and octahedral B sites [68]. Generally, for spinel ferrite, the saturation magnetization is influenced by magnetic super exchange interaction of cations between tetrahedral A and octahedral B sites. The iron ions from B-sites are the dominant contributor to the magnetization. When lanthanum ions are introduced at the expense of iron ion, some of the Fe^3+^ migrates from B- to A-sites in view of the site preferences for different ions which lead to the increase of Fe^3+^ concentration at A-sites. The extended radius of lanthanum ion and the small tetrahedral A site gap pressurize the La^3+^ to enter the octahedral B site. The B-B exchange interaction at the octahedral site reduces as a result of decreasing iron ion concentration which will eventually reduce the A-B super-exchange interaction too. The weakening of A-O-B super exchange interactions attributes to the decrease of saturation magnetization. Moreover, La^3+^ ion has no electrons in 4f orbit which makes it paramagnetic. Therefore, when the paramagnetic La^3+^ ion replaces the Fe^3+^ ion at B-site it leads to the decrease in the net overall magnetic moment. Further, the substitution of paramagnetic La^3+^ ion leads to distortion of the spinel lattice thus changes the spin magnetic moment between the A-B sublattices into nonlinear antiferromagnetic coupling [15, 69, 70]. The above discussion confirmed the reduction in the magnetization of prepared ferrite nanocrystals samples at lanthanum ion concentration at above X ≥ 0.06.

Generally, the coercivity value portrays the strength of the magnetic field that is required to surpass the anisotropy barrier and allow the magnetization of the Ni_0.5_Zn_0.5_La_x_Fe_1-x_O_4_ nanoparticles following the magnetic field orientation. From the Fig 3 it can be observed that the coercivity and remanence values have the same trend with respect to the lattice constants (Å) where these values are not consistent with the increase in La^3+^ ion concentration. The coercivity governs by factors such as magneto crystalline anisotropy, microstrain, magnetic particle morphology, size distribution, shape anisotropy, and magnetic content [68, 71].

On the basis of the above explanation, the predicted distribution of ions for the system under investigation is proposed in the following outline:

Generally, Ni^2+^ ion has a strong tendency to occupy octahedral site (B-site), and Zn^2+^ ion occupy a tetrahedral site (A-site) only. On the contrary, Fe^3+^ ions are distributed between the two sites. Therefore, the cation distribution of Ni-Zn spinel ferrite can be represented by the following formula [4].

$$\left(Zn_{x}^{2+},Fe_{1-x}^{3+}\right)\left[Ni_{1-x}^{2+},Fe_{1+x}^{3+}\right]\;O_{4}^{2-}$$ (3)

where the brackets () and [] demonstrates A and B sites respectively. The preference of ions of basic composition Ni_0.5_Zn_0.5_Fe_2_O_4_ into the A and B sites are influenced by theoretical formula as follows:

$$\left(Zn^{2+}{}_{0.5}\;Fe^{3+}{}_{0.42}\right)\;\left[Ni^{2+}{}_{0.5}Fe^{3+}{}_{1.58}\right]O^{2\text{-}}{}_{4}$$ (4)

Rare earth ions are known to occupy the octahedral B-sites. Therefore, lanthanum ion has an initial preference for the octahedral site, where it replaced Fe3+ ions in the B-sites as per the formula below.

$$\left(Zn^{2+}{}_{0.5}\;Fe^{3+}{}_{0.42}\right)\;\left[Ni^{2+}{}_{0.5}La^{3+}{}_{x}Fe^{3+}{}_{1.58\text{-}x}\right]O^{2\text{-}}{}_{4}$$ (5)

The total magnetization decreases with an increasing La^3+^ concentration, thus it proves that the substitution of paramagnetic lanthanum ion into ferromagnetic iron ions in spinel does not improve the magnetic properties of the synthesized nanoparticles [72].

## Conclusion

The present study demonstrates the effect of La^3+^ substitution for Fe^3+^ in Ni-Zn ferrite nanocrystals morphology, structure, and magnetic properties. The substitution of lanthanum ion was found to affect the structure, morphology and magnetic properties of Ni-Zn ferrite nanocrystals. Structural analysis of La^3+^ substituted nanoparticles showed a cubic spinel ferrite with the presence of small peaks attributed to secondary phase due to La^3+^ substitution. The crystallite size of prepared nanoparticles has increased with an increase in lanthanum ion concentration of X ≤ 0.06 due to large ionic radii of La^3+^ ion. Further, the saturation magnetization of substituted samples decreases with an increase of lanthanum ion concentration. This is due to dilution of the net magnetic field by paramagnetic La^3+^ ion. While the spectroscopic analysis of these materials demonstrates the changes in the frequency intensities due to the addition of La^3+^ ion which affects the bond length between Fe^3+^- O^2-^ at both the A and B sites. Finally, the observed results have indicated that the rare earth lanthanum ions prefer to occupy the octahedral B-site of spinel ferrites.

## References

[1] Sellmyer DJ, Luo CP, Qiang Y, Liu JP. Chapter 7—Magnetism of nanophase composite films A2—Nalwa, Hari Singh Handbook of Thin Films. Burlington: Academic Press; 2002 p. 337–74.

[2] Patil R, Delekar S, Mane D, Hankare P. Synthesis, structural and magnetic properties of different metal ion substituted nanocrystalline zinc ferrite. Results in Physics. 2013;3:129–33.

[3] Caizer C, Stefanescu M. Magnetic characterization of nanocrystalline Ni–Zn ferrite powder prepared by the glyoxylate precursor method. Journal of Physics D: Applied Physics. 2002;35(23):3035.

[4] Shirsath SE, Toksha BG, Kadam RH, Patange SM, Mane DR, Jangam GS, et al Doping effect of Mn^2+^ on the magnetic behavior in Ni–Zn ferrite nanoparticles prepared by sol–gel auto-combustion. Journal of Physics and Chemistry of Solids. 2010;71(12):1669–75.

[5] Najmoddin N, Beitollahi A, Kavas H, Mohseni SM, Rezaie H, Åkerman J, et al XRD cation distribution and magnetic properties of mesoporous Zn-substituted CuFe_2_O_4_. Ceramics International. 2014;40(2):3619–25.

[6] Ahmad I, Abbas T, Islam M, Maqsood A. Study of cation distribution for Cu–Co nanoferrites synthesized by the sol–gel method. Ceramics International. 2013;39(6):6735–41.

[7] Hussain A, Abbas T, Niazi SB. Preparation of Ni_1− x_Mn_x_Fe_2_O_4_ ferrites by sol–gel method and study of their cation distribution. Ceramics International. 2013;39(2):1221–5.

[8] Kavas H, Baykal A, Toprak MS, Köseoğlu Y, Sertkol M, Aktaş B. Cation distribution and magnetic properties of Zn doped NiFe_2_O_4_ nanoparticles synthesized by PEG-assisted hydrothermal route. Journal of Alloys and Compounds. 2009;479(1):49–55.

[9] Rani R, Sharma S, Pirota K, Knobel M, Thakur S, Singh M. Effect of zinc concentration on the magnetic properties of cobalt–zinc nanoferrite. Ceramics International. 2012;38(3):2389–94.

[10] Ahmed M, Ateia E, Salah L, El-Gamal A. Structural and electrical studies on La^3+^ substituted Ni–Zn ferrites. Materials chemistry and physics. 2005;92(2):310–21.

[11] Ahmed TT, Rahman IZ, Rahman MA. Study on the properties of the copper substituted NiZn ferrites. Journal of Materials Processing Technology. 2004;153–154:797–803.

[12] Sláma J, Grusková A, Ušáková M, Ušák E, Dosoudil R. Contribution to analysis of Cu-substituted NiZn ferrites. Journal of Magnetism and Magnetic Materials. 2009;321(19):3346–51.

[13] Sattar AA, El-Sayed HM, El-Shokrofy KM, El-Tabey MM. Effect of manganese substitution on the magnetic properties of nickel-zinc ferrite. Journal of Materials Engineering and Performance. 2005;14(1):99–103.

[14] Peng Z, Fu X, Ge H, Fu Z, Wang C, Qi L, et al Effect of Pr^3+^ doping on magnetic and dielectric properties of Ni–Zn ferrites by “one-step synthesis”. Journal of Magnetism and Magnetic Materials. 2011;323(20):2513–8.

[15] Wang Y, Wu X, Zhang W, Chen W. Synthesis and electromagnetic properties of La-doped Ni–Zn ferrites. Journal of Magnetism and Magnetic Materials. 2016;398:90–5.

[16] Shinde TJ, Gadkari AB, Vasambekar PN. Influence of Nd^3+^ substitution on structural, electrical and magnetic properties of nanocrystalline nickel ferrites. Journal of Alloys and Compounds. 2012;513:80–5.

[17] Huq M, Saha D, Ahmed R, Mahmood Z. Ni-Cu-Zn Ferrite Research: A Brief Review. Journal of Scientific Research. 2013;5(2):215–34.

[18] Krishnamurthy N, Gupta CK. Extractive Metallurgy of Rare Earths: CRC Press; 2004.

[19] Schwartz M. Encyclopedia of Materials, Parts and Finishes, Second Edition: CRC Press; 2002.

[20] Humphries M. Rare Earth Elements: The Global Supply Chain: DIANE Publishing Company; 2010.

[21] Chu S. Critical Materials Strategy: DIANE Publishing Company; 2011.

[22] Survey G. Minerals Yearbook, 2008, V. 1, Metals and Minerals: U.S. Government Printing Office; 2011.

[23] Pervaiz E, Gul I, editors. Influence of rare earth (Gd^3+^) on structural, gigahertz dielectric and magnetic studies of cobalt ferrite Journal of Physics: Conference Series; 2013: IOP Publishing.

[24] Xavier S, Thankachan S, Jacob BP, Mohammed EM. Effect of Samarium Substitution on the Structural and Magnetic Properties of Nanocrystalline Cobalt Ferrite. Journal of Nanoscience. 2013;2013:7.: 10.1155/2013/524380.

[25] Jacob BP, Thankachan S, Xavier S, Mohammed E. Effect of Tb^3+^ substitution on structural, electrical and magnetic properties of sol–gel synthesized nanocrystalline nickel ferrite. Journal of Alloys and Compounds. 2013;578:314–9.

[26] Zhou B, Zhang Y-W, Liao C-S, Yan C-H, Chen L-Y, Wang S-Y. Rare-earth-mediated magnetism and magneto-optical Kerr effects in nanocrystalline CoFeMn_0.9_RE_0.1_O_4_ thin films. Journal of magnetism and magnetic materials. 2004;280(2):327–33.

[27] Satalkar M, Kane SN. On structural studies and cation distribution of La added Zn-Ni-Mg-Cu spinel nano ferrite. Journal of Physics: Conference Series. 2016;755(1):012047.

[28] Dar MA, Shah J, Siddiqui W, Kotnala R. Study of structure and magnetic properties of Ni–Zn ferrite nano-particles synthesized via co-precipitation and reverse micro-emulsion technique. Applied Nanoscience. 2014;4(6):675–82.

[29] Köseoğlu Y, Bay M, Tan M, Baykal A, Sözeri H, Topkaya R, et al Magnetic and dielectric properties of Mn_0.2_Ni_0.8_Fe_2_O_4_ nanoparticles synthesized by PEG-assisted hydrothermal method. Journal of Nanoparticle Research. 2011;13(5):2235–44.

[30] Yang H, Zhang X, Ao W, Qiu G. Formation of NiFe_2_O_4_ nanoparticles by mechanochemical reaction. Materials Research Bulletin. 2004;39(6):833–7.

[31] Jing J, Liangchao L, Feng X. Structural analysis and magnetic properties of Gd-doped Li-Ni ferrites prepared using rheological phase reaction method. Journal of Rare Earths. 2007;25(1):79–83.

[32] Wu X, Wu W, Qin L, Wang K, Ou S, Zhou K, et al Structure and magnetic properties evolution of nickel–zinc ferrite with lanthanum substitution. Journal of Magnetism and Magnetic Materials. 2015;379:232–8.

[33] Valenzuela R. Magnetic ceramics: Cambridge University Press; 2005.

[34] George M, Nair SS, John AM, Joy P, Anantharaman M. Structural, magnetic and electrical properties of the sol-gel prepared Li_0.5_Fe_2.5_O_4_ fine particles. Journal of Physics D: Applied Physics. 2006;39(5):900.

[35] Guan BH, Zahari MH, Chuan LK, editors. Structural and Magnetic Properties of Ni_0.5_Zn_0.5_Fe_2_O_4_ Synthesized through the Sol-Gel Method Applied Mechanics and Materials; 2014: Trans Tech Publ.

[36] Gao P, Rebrov EV, Verhoeven TM, Schouten JC, Kleismit R, Kozlowski G, et al Structural investigations and magnetic properties of sol-gel Ni_0.5_Zn_0.5_Fe_2_O_4_ thin films for microwave heating. Journal of Applied Physics. 2010;107(4):044317.

[37] Nair KM, Jia Q, Priya S, Society AC. Advances and Applications in Electroceramics: Ceramic Transactions: Wiley; 2011.

[38] Zahi S, Hashim M, Daud AR. Synthesis, magnetic properties and microstructure of Ni–Zn ferrite by sol–gel technique. Journal of Magnetism and Magnetic Materials. 2007;308(2):177–82.

[39] Zahi S. Synthesis, Permeability and Microstructure of the Optimal Nickel-Zinc Ferrites by Sol-Gel Route. Journal of Electromagnetic Analysis and Applications. 2010;2(1):56–62.

[40] Bugad R, Mane T, Navale B, Thombare J, Babar A, Karche B. Structural, morphological and compositional properties of La^3+^ substituted Mg–Zn ferrite interlocked nanoparticles by co-precipitation method. Journal of Materials Science: Materials in Electronics.1–7.

[41] Stergiou C, Litsardakis G, editors. Structural and magnetic properties of yttrium and lanthanum-doped Ni-Co and Ni-Co-Zn spinel ferrites. AIP Conference Proceedings; 2014.

[42] Wang Y, Xu F, Li L, Liu H, Qiu H, Jiang J. Magnetic properties of La-substituted Ni–Zn–Cr ferrites via rheological phase synthesis. Materials Chemistry and Physics. 2008;112(3):769–73.

[43] Al Angari Y. Magnetic properties of La-substituted NiFe_2_O_4_ via egg-white precursor route. Journal of Magnetism and Magnetic Materials. 2011;323(14):1835–9.

[44] K Rama K, K Vijaya K, Dachepalli R. Structural and electrical conductivity studies in nickel-zinc ferrite. Advances in Materials physics and chemistry. 2012;2012.

[45] Gao F, Qin G, Li Y, Jiang Q, Luo L, Zhao K, et al One-pot synthesis of La-doped SnO_2_ layered nanoarrays with an enhanced gas-sensing performance toward acetone. RSC Advances. 2016;6(13):10298–310.

[46] Awati V, Rathod S, Shirsath SE, Mane ML. Fabrication of Cu^2+^ substituted nanocrystalline Ni–Zn ferrite by solution combustion route: Investigations on structure, cation occupancy and magnetic behavior. Journal of Alloys and Compounds. 2013;553:157–62.

[47] Singh N, Agarwal A, Sanghi S, Singh P. Effect of magnesium substitution on dielectric and magnetic properties of Ni–Zn ferrite. Physica B: Condensed Matter. 2011;406(3):687–92.

[48] Al Angari YM. Magnetic properties of La-substituted NiFe_2_O_4_ via egg-white precursor route. Journal of Magnetism and Magnetic Materials. 2011;323(14):1835–9.

[49] Rathod SM, Bhosale SS, Zagade PK, Pawar DB, Shinde AB. Synthesis and Characterizationof La3+ Doped Ni NanoFerriteby Sol-Gel Method.

[50] Chaudhari V, Shirsath SE, Mane M, Kadam R, Shelke S, Mane D. Crystallographic, magnetic and electrical properties of Ni_0.5_Cu_0.25_Zn_0.25_La_x_Fe_2−x_O_4_ nanoparticles fabricated by sol–gel method. Journal of Alloys and Compounds. 2013;549:213–20.

[51] Kulkarni V, Bhujbal M, Rathod S. Influence of La3+ Doped Ni-Co Nanoferrite and Magnetic Properties by Sol-Gel Auto Combustion Method.

[52] Anupama MK, Rudraswamy B. Effect of Gd^3+^—Cr^3+^ ion substitution on the structural, electrical and magnetic properties of Ni—Zn ferrite nanoparticles. IOP Conference Series: Materials Science and Engineering. 2016;149(1):012194.

[53] Rezlescu N, Rezlescu E, Popa P, Rezlescu L. Effects of rare-earth oxides on physical properties of Li–Zn ferrite. Journal of alloys and compounds. 1998;275:657–9.

[54] Masoudpanah S, Ebrahimi SS, Derakhshani M, Mirkazemi S. Structure and magnetic properties of La substituted ZnFe_2_O_4_ nanoparticles synthesized by sol–gel autocombustion method. Journal of Magnetism and Magnetic Materials. 2014;370:122–6.

[55] Chaudhari V, Shirsath SE, Mane ML, Kadam RH, Shelke SB, Mane DR. Crystallographic, magnetic and electrical properties of Ni_0.5_Cu_0.25_Zn_0.25_La_x_Fe_2−x_O_4_ nanoparticles fabricated by sol–gel method. Journal of Alloys and Compounds. 2013;549:213–20.

[56] Gadkari A, Shinde T, Vasambekar P. Synthesis, characterization and magnetic properties of La^3+^ added Mg–Cd ferrites prepared by oxalate co-precipitation method. Journal of Alloys and Compounds. 2011;509(3):966–72.

[57] Ali I, Ahmad M, Islam M, Awan M. Substitution effects of La^3+^ ions on the structural and magnetic properties of Co_2_Y hexaferrites synthesized by sol–gel autocombustion method. Journal of sol-gel science and technology. 2013;68(1):141–9.

[58] Torkian S, Ghasemi A, ShojaRazavi R, Tavoosi M. Structural and Magnetic Properties of High Coercive Al-Substituted Strontium Hexaferrite Nanoparticles. Journal of Superconductivity and Novel Magnetism. 2016;29(6):1627–40.

[59] Ateia EE, Abdelatif G, Ahmed M, Mahmoud MAA. Effect of Different Gd^3+^ Ion Content on the Electric and Magnetic Properties of Lithium Antimony Ferrite. Journal of Inorganic and Organometallic Polymers and Materials. 2016;26(1):81–90.

[60] Sanpo N, Wen C, Berndt CC, Wang J. Antibacterial properties of spinel ferrite nanoparticles. 2013.

[61] Jiang J, Yang Y-M, Li L-C. Synthesis and magnetic properties of lanthanum-substituted lithium–nickel ferrites via a soft chemistry route. Physica B: Condensed Matter. 2007;399(2):105–8.

[62] Gaber A, Abdel-Rahim M, Abdel-Latief A, Abdel-Salam MN. Influence of calcination temperature on the structure and porosity of nanocrystalline SnO_2_ synthesized by a conventional precipitation method. Int J Electrochem Sci. 2014;9(1):81–95.

[63] Gabal MA, Asiri AM, AlAngari YM. On the structural and magnetic properties of La-substituted NiCuZn ferrites prepared using egg-white. Ceramics International. 2011;37(7):2625–30.

[64] Ali AI, Ahmed MA, Okasha N, Hammam M, Son JY. Effect of the La^3+^ ions substitution on the magnetic properties of spinal Li-Zn-ferrites at low temperature. Journal of Materials Research and Technology. 2013;2(4):356–61.

[65] Sridhar R, Dachepalli R, K Vijaya K. Synthesis and characterization of copper substituted nickel nano-ferrites by citrate-gel technique. Advances in Materials Physics and Chemistry. 2012;2012.

[66] Kumar L, Kar M. Effect of La^3+^ substitution on the structural and magnetocrystalline anisotropy of nanocrystalline cobalt ferrite (CoFe_2−x_La_x_O_4_). Ceramics International. 2012;38(6):4771–82.

[67] Kodama RH, Berkowitz AE, McNiff E Jr, Foner S. Surface spin disorder in NiFe_2_O_4_ nanoparticles. Physical Review Letters. 1996;77(2):394 doi: 10.1103/PhysRevLett.77.394 10062440 10.1103/PhysRevLett.77.394

[68] Shirsath SE, Toksha B, Kadam R, Patange S, Mane D, Jangam GS, et al Doping effect of Mn^2+^ on the magnetic behavior in Ni–Zn ferrite nanoparticles prepared by sol–gel auto-combustion. Journal of Physics and Chemistry of Solids. 2010;71(12):1669–75.

[69] Jacob J, Khadar MA. Investigation of mixed spinel structure of nanostructured nickel ferrite. Journal of applied physics. 2010;107(11):114310.

[70] Gabal MA, Asiri AM, AlAngari Y. On the structural and magnetic properties of La-substituted NiCuZn ferrites prepared using egg-white. Ceramics International. 2011;37(7):2625–30.

[71] Chang-Min X, Ji-Rong S, Deng-Jing W, Guan-Juan L, Hong-Wei Z, Bao-Gen S. Dependence of the coercivity of La_0.67_Ca_0.33_MnO_3_ films on substrate and thickness. Chinese Physics. 2005;14(3):604.

[72] Šoka M, Ušáková M, Ušák E. Studies of rare-earth substituted nickel zinc ferrites. Journal of Electrical Engineering. 2012;63(7s):83–5.

